# Supplementary material for: Reduced midbrain raphe echogenicity in patients with fibromyalgia syndrome
Source: PLoS One. 2022 Nov 17;17(11):e0277316. doi: 10.1371/journal.pone.0277316 (PMC9671316; doi:10.1371/journal.pone.0277316)
Supplement: S1 Table — (DOCX) [file pone.0277316.s001.docx]

**Supplementary Table 1: Spearman correlation analysis in the group of patients with fibromyalgia syndrome.**

| **Correlations** | | | | | | | | | | |
| --- | --- | --- | --- | --- | --- | --- | --- | --- | --- | --- |
|  | | | Raphe (quantitative) | NPSI sum score | GCPS current pain | ADS | Pain duration | FIQ | WPI | SSS |
| Spearman‘s rho | Raphe (quantitative) | Correlation Coefficient | 1.000 | -.183 | -.066 | -.057 | -.140 | .028 | -.036 | .344^*^ |
|  |  | Sig. (2-tailed) | . | .224 | .667 | .709 | .369 | .857 | .811 | .019 |
|  |  | N | 46 | 46 | 45 | 46 | 43 | 44 | 46 | 46 |
|  | NPSI sum score | Correlation Coefficient | -.183 | 1.000 | .552^**^ | .389^**^ | -.026 | .497^**^ | .349^*^ | -.060 |
|  |  | Sig. (2-tailed) | .224 | . | <.001 | .004 | .858 | <.001 | .011 | .672 |
|  |  | N | 46 | 52 | 51 | 52 | 49 | 50 | 52 | 52 |
|  | GCPS current pain | Correlation Coefficient | -.066 | .552^**^ | 1.000 | .308^*^ | -.148 | .574^**^ | .244 | .237 |
|  |  | Sig. (2-tailed) | .667 | <.001 | . | .028 | .315 | <.001 | .084 | .094 |
|  |  | N | 45 | 51 | 51 | 51 | 48 | 49 | 51 | 51 |
|  | ADS | Correlation Coefficient | -.057 | .389^**^ | .308^*^ | 1.000 | -.278 | .586^**^ | -.064 | .299^*^ |
|  |  | Sig. (2-tailed) | .709 | .004 | .028 | . | .053 | <.001 | .653 | .031 |
|  |  | N | 46 | 52 | 51 | 52 | 49 | 50 | 52 | 52 |
|  | Pain duration | Correlation Coefficient | -.140 | -.026 | -.148 | -.278 | 1.000 | -.204 | .187 | -.153 |
|  |  | Sig. (2-tailed) | .369 | .858 | .315 | .053 | . | .169 | .199 | .294 |
|  |  | N | 43 | 49 | 48 | 49 | 49 | 47 | 49 | 49 |
|  | FIQ | Correlation Coefficient | .028 | .497^**^ | .574^**^ | .586^**^ | -.204 | 1.000 | .160 | .346^*^ |
|  |  | Sig. (2-tailed) | .857 | <.001 | <.001 | <.001 | .169 | . | .268 | .014 |
|  |  | N | 44 | 50 | 49 | 50 | 47 | 50 | 50 | 50 |
|  | WPI | Correlation Coefficient | -.036 | .349^*^ | .244 | -.064 | .187 | .160 | 1.000 | .062 |
|  |  | Sig. (2-tailed) | .811 | .011 | .084 | .653 | .199 | .268 | . | .659 |
|  |  | N | 46 | 52 | 51 | 52 | 49 | 50 | 53 | 53 |
|  | SSS | Correlation Coefficient | .344^*^ | -.060 | .237 | .299^*^ | -.153 | .346^*^ | .062 | 1.000 |
|  |  | Sig. (2-tailed) | .019 | .672 | .094 | .031 | .294 | .014 | .659 | . |
|  |  | N | 46 | 52 | 51 | 52 | 49 | 50 | 53 | 53 |
| *. Correlation is significant at the 0.05 level (2-tailed). | | | | | | | | | | |
| **. Correlation is significant at the 0.01 level (2-tailed). | | | | | | | | | | |

Abbreviations: ADS: „Allgemeine Depressionsskala“, FIQ: Fibromyalgia Impact Questionnaire, GCPS: Graded Chronic Pain Scale, NPSI: Neuropathic Pain Symptom Inventory, SSS: Symptom Severity Score, WPI: Widespread Pain Index.
