# Supplementary material for: Reduced midbrain raphe echogenicity in patients with fibromyalgia syndrome
Source: PLoS One. 2022 Nov 17;17(11):e0277316. doi: 10.1371/journal.pone.0277316 (PMC9671316; doi:10.1371/journal.pone.0277316)
Supplement: S2 Table — (DOCX) [file pone.0277316.s002.docx]

**Supplementary Table 2: Spearman correlation analysis in the group of patients with depression and physical pain.**

| **Correlations** | | | | | | | | | | |
| --- | --- | --- | --- | --- | --- | --- | --- | --- | --- | --- |
|  | | | Raphe (quantitative) | NPSI sum score | GCPS current pain | ADS | Pain duration | FIQ | WPI | SSS |
| Spearman‘s rho | Raphe (quantitative) | Correlation Coefficient | 1.000 | -.058 | .510 | .294 | -.130 | .330 | . | . |
|  |  | Sig. (2-tailed) | . | .851 | .075 | .329 | .672 | .271 | . | . |
|  |  | N | 13 | 13 | 13 | 13 | 13 | 13 | 0 | 0 |
|  | NPSI sum score | Correlation Coefficient | -.058 | 1.000 | .043 | .044 | -.078 | .219 | . | . |
|  |  | Sig. (2-tailed) | .851 | . | .889 | .886 | .801 | .472 | . | . |
|  |  | N | 13 | 13 | 13 | 13 | 13 | 13 | 0 | 0 |
|  | GCPS current pain | Correlation Coefficient | .510 | .043 | 1.000 | .379 | .119 | .346 | . | . |
|  |  | Sig. (2-tailed) | .075 | .889 | . | .201 | .699 | .247 | . | . |
|  |  | N | 13 | 13 | 13 | 13 | 13 | 13 | 0 | 0 |
|  | ADS | Correlation Coefficient | .294 | .044 | .379 | 1.000 | -.305 | .555^*^ | . | . |
|  |  | Sig. (2-tailed) | .329 | .886 | .201 | . | .311 | .049 | . | . |
|  |  | N | 13 | 13 | 13 | 13 | 13 | 13 | 0 | 0 |
|  | Pain duration | Correlation Coefficient | -.130 | -.078 | .119 | -.305 | 1.000 | -.132 | . | . |
|  |  | Sig. (2-tailed) | .672 | .801 | .699 | .311 | . | .668 | . | . |
|  |  | N | 13 | 13 | 13 | 13 | 13 | 13 | 0 | 0 |
|  | FIQ | Correlation Coefficient | .330 | .219 | .346 | .555^*^ | -.132 | 1.000 | . | . |
|  |  | Sig. (2-tailed) | .271 | .472 | .247 | .049 | .668 | . | . | . |
|  |  | N | 13 | 13 | 13 | 13 | 13 | 13 | 0 | 0 |
|  | WPI | Correlation Coefficient | . | . | . | . | . | . | . | . |
|  |  | Sig. (2-tailed) | . | . | . | . | . | . | . | . |
|  |  | N | 0 | 0 | 0 | 0 | 0 | 0 | 0 | 0 |
|  | SSS | Correlation Coefficient | . | . | . | . | . | . | . | . |
|  |  | Sig. (2-tailed) | . | . | . | . | . | . | . | . |
|  |  | N | 0 | 0 | 0 | 0 | 0 | 0 | 0 | 0 |
| *. Correlation is significant at the 0.05 level (2-tailed). | | | | | | | | | | |

Abbreviations: ADS: „Allgemeine Depressionsskala“, FIQ: Fibromyalgia Impact Questionnaire, GCPS: Graded Chronic Pain Scale, NPSI: Neuropathic Pain Symptom Inventory, SSS: Symptom Severity Score, WPI: Widespread Pain Index.
