# Supplementary material for: Reduced midbrain raphe echogenicity in patients with fibromyalgia syndrome
Source: PLoS One. 2022 Nov 17;17(11):e0277316. doi: 10.1371/journal.pone.0277316 (PMC9671316; doi:10.1371/journal.pone.0277316)
Supplement: Attachment — Submitted filename: Reply_111022.docx [file pone.0277316.s003.docx]

When submitting your revision, we need you to address these additional requirements.

1. Please ensure that your manuscript meets PLOS ONE's style requirements, including those for file naming. The PLOS ONE style templates can be found at 
<https://journals.plos.org/plosone/s/file?id=wjVg/PLOSOne_formatting_sample_main_body.pdf> and 
<https://journals.plos.org/plosone/s/file?id=ba62/PLOSOne_formatting_sample_title_authors_affiliations.pdf>

Done.

2. Thank you for stating in your Funding Statement: 
"Parts of the study was supported by the Else Kröner-Fresenius-Stiftung (N.Ü.: 2014_A129). N.Ü. was funded by Deutsche Forschungsgemeinschaft (DFG; UE171/15-1)."

Please provide an amended statement that declares *all* the funding or sources of support (whether external or internal to your organization) received during this study, as detailed online in our guide for authors at <http://journals.plos.org/plosone/s/submit-now>.  Please also include the statement “There was no additional external funding received for this study.” in your updated Funding Statement. 
Please include your amended Funding Statement within your cover letter. We will change the online submission form on your behalf.

Done.

3. Thank you for stating the following financial disclosure: 
"Parts of the study was supported by the Else Kröner-Fresenius-Stiftung (N.Ü.: 2014_A129). N.Ü. was funded by Deutsche Forschungsgemeinschaft (DFG; UE171/15-1)."
 
Please state what role the funders took in the study. If the funders had no role, please state: ""The funders had no role in study design, data collection and analysis, decision to publish, or preparation of the manuscript."" 
If this statement is not correct you must amend it as needed. 
Please include this amended Role of Funder statement in your cover letter; we will change the online submission form on your behalf.

Done.

4. In your Data Availability statement, you have not specified where the minimal data set underlying the results described in your manuscript can be found. PLOS defines a study's minimal data set as the underlying data used to reach the conclusions drawn in the manuscript and any additional data required to replicate the reported study findings in their entirety. All PLOS journals require that the minimal data set be made fully available. For more information about our data policy, please see <http://journals.plos.org/plosone/s/data-availability>.

Upon re-submitting your revised manuscript, please upload your study’s minimal underlying data set as either Supporting Information files or to a stable, public repository and include the relevant URLs, DOIs, or accession numbers within your revised cover letter. For a list of acceptable repositories, please see <http://journals.plos.org/plosone/s/data-availability#loc-recommended-repositories>. Any potentially identifying patient information must be fully anonymized.

Important: If there are ethical or legal restrictions to sharing your data publicly, please explain these restrictions in detail. Please see our guidelines for more information on what we consider unacceptable restrictions to publicly sharing data: <http://journals.plos.org/plosone/s/data-availability#loc-unacceptable-data-access-restrictions>. Note that it is not acceptable for the authors to be the sole named individuals responsible for ensuring data access.

We will update your Data Availability statement to reflect the information you provide in your cover letter.

Done.

5. Please include captions for your Supporting Information files at the end of your manuscript, and update any in-text citations to match accordingly. Please see our Supporting Information guidelines for more information: <http://journals.plos.org/plosone/s/supporting-information>.

Done.

**Additional Editor Comments:**

This is an interesting study, but the sample size needs to be increased for the results to be meaningful. Please revise your manuscript carefully according to the reviewers' comments.

We have followed the Editor`s suggestion and have added data of further 23 subjects. Respective modifications were made in the Methods and Results sections of our revised manuscript.

[Note: HTML markup is below. Please do not edit.]

Reviewers' comments:

Reviewer's Responses to Questions

**Comments to the Author**

1. Is the manuscript technically sound, and do the data support the conclusions?

The manuscript must describe a technically sound piece of scientific research with data that supports the conclusions. Experiments must have been conducted rigorously, with appropriate controls, replication, and sample sizes. The conclusions must be drawn appropriately based on the data presented.

Reviewer #1: Partly

Reviewer #2: Yes

2. Has the statistical analysis been performed appropriately and rigorously?

Reviewer #1: N/A

Reviewer #2: Yes

3. Have the authors made all data underlying the findings in their manuscript fully available?

The [PLOS Data policy](http://www.plosone.org/static/policies.action#sharing) requires authors to make all data underlying the findings described in their manuscript fully available without restriction, with rare exception (please refer to the Data Availability Statement in the manuscript PDF file). The data should be provided as part of the manuscript or its supporting information, or deposited to a public repository. For example, in addition to summary statistics, the data points behind means, medians and variance measures should be available. If there are restrictions on publicly sharing data—e.g. participant privacy or use of data from a third party—those must be specified.

Reviewer #1: Yes

Reviewer #2: Yes

4. Is the manuscript presented in an intelligible fashion and written in standard English?

PLOS ONE does not copyedit accepted manuscripts, so the language in submitted articles must be clear, correct, and unambiguous. Any typographical or grammatical errors should be corrected at revision, so please note any specific errors here.

Reviewer #1: Yes

Reviewer #2: Yes

5. Review Comments to the Author

Please use the space provided to explain your answers to the questions above. You may also include additional comments for the author, including concerns about dual publication, research ethics, or publication ethics. (Please upload your review as an attachment if it exceeds 20,000 characters)

Reviewer #1: It is a very interesting study that use a new classification( off ) of midbrain raphe echo-intensity, and also has new application of TCS for FMS.
There are still some issues need to be clarified.

1. How to diagnose FMS

The diagnosis of FMS was made according to the Criteria of the American College of Rheumatology 1990, 2010, and the German S3 evidence-based FMS guidelines.^1-3^ This information was already given in the first paragraph of the Methods section of our manuscript.

2. why only females included in this study?

We have revised our manuscript and have now also included men. Respective information are given in the Methods and Results section of our revised manuscript.

3.The number of cases is relatively small, so the conclusions drawn are not completely reliable, It is recommended to increase the sample size.

We have followed the Reviewer´s suggestion and have increased our study cohort by data of further 23 subjects. Respective modifications were made in the Methods and Results sections of our manuscript. In addition, we performed a second reading of all midbrain sonograms by an independent reader, which yielded a high interrater reliability of raphe echogenicity assessments (Cohen’s kappa=0.85). We included this information in the Methods and Results sections of our revised manuscript.

4.In this study, the ADS score of the depression + pain group was significantly higher than that of the FMS group, so a Spearman correlation analysis could not draw a true correlation between midbrain raphe echo-intensity and the existence of confounding factors.

ADS scores were >16 in both groups indicating clinical significance.^4^ A higher score in the depression group is plausible since here, depression is the main disease while in FMS, depressive symptoms accompany pain. We had pointed out this aspect in the Discussion section of our original manuscript:

“*Interestingly, sonography results did not correlate with the presence and severity of depressive symptoms as quantified using the ADS sum score which was higher in patients with depression than in FMS patients (Table 1)*.”

Hence, we agree that cross-correlations would not lead to reliable data and have refrained from calculating these. Data on correlation analysis presented in our manuscript merely remain within each study group. We have re-worded the respective passage in the Discussion section of our revised manuscript (please see page 8):

“Interestingly, sonography results did not correlate with the presence and severity of depressive symptoms in each study group as quantified using the ADS sum score which was higher in patients with depression than in FMS patients (Table 1). Hence, cross-correlations are not possible.”

5.Many studies revealed that reduce midbrain raphe echo-intensity was associated with depression in a series of disease.(33912243，30131761), why here no correlation was found between midbrain raphe echo-intensity and depression + pain group? The reason in the discussion is not sufficient, which need to be discussed in more depth.

Thank you for this comment.

33912243: This study primarily investigated patients with migraine and additional depressive symptoms using the HAM-D questionnaire. No psychiatric interview and clinical investigation was performed; hence, the diagnosis of depression cannot be made merely using one questionnaire. In contrast, we investigated patients with a confirmed psychiatric diagnosis of depression.

30131761: This study primarily examined patients with Parkinson`s disease or essential tremor. No psychiatric diagnosis of depression was made. Merely data of the BDI questionnaire were used to screen for depressive symptoms and none of the investigated patients received anti-depressant treatment suggesting low intensity of symptoms, if any.

We believe that great caution is necessary when cross-comparing studies reporting data of patients with a psychiatrically confirmed depression with those of patients suffering from a primarily other disease but showing hints for depressive symptoms merely assessed with questionnaires. We have added a respective passage in the Discussion section of our revised manuscript (please see page 9):

“As for the seemingly contradictory data in our study to published literature regarding reduced midbrain echogenicity in patients with depressive symptoms it is of note that we investigated patients with a psychiatrically confirmed diagnosis of depression, while in other studies, mainly patients with another primary disease such as migraine^5^ or Parkinson`s disease^6^ were studied who additionally had depressive symptoms. Hence, data may differ between patients with depression and those with mere depressive symptoms.”

Reviewer #2: This is an interesting research which is novel and has not been done in the past.
I have just a couple of minor comments:

1. in the abstract the conclusion and results looks similar, it is recommended to present a few sentences regarding the pathophysiology of the findings. I mean it is better to write briefly about the value of the findings which are presented in result section.

We have followed the Reviewer`s suggestion and have rephrased our Abstract conclusions accordingly:

“We found reduced echogenicity of the midbrain raphe area in patients with FMS and in patients with depression and physical pain, independent of the presence or severity of pain, FMS, and depressive symptoms. Further exploration of this sonographic finding is necessary before this objective technique may enter diagnostic algorithms in FMS and depression.”

2. In the method please mention the criteria you selected patients in FMS group.

The FMS diagnosis was made according to the Criteria of the American College of Rheumatology 1990, 2010, and the German S3 evidence-based guidelines.^1-3^ This information was already given in the first paragraph of the Methods section of our manuscript.

3. Also explain in the text about the pain group. How different they were from FMS group? Which pain they were suffering from?

We have followed the Reviewer`s suggestion and have added further details on the pain phenotype of the depression + pain group. Data were implemented in the Results section of our revised manuscript.

4. It is better that the text be revised regarding the writing method, there are long sentences which make reading difficult.

Thank you. We have carefully revised our manuscript accordingly.

6. PLOS authors have the option to publish the peer review history of their article ([what does this mean?](https://journals.plos.org/plosone/s/editorial-and-peer-review-process#loc-peer-review-history)). If published, this will include your full peer review and any attached files.


If you choose “no”, your identity will remain anonymous but your review may still be made public.


**Do you want your identity to be public for this peer review?** For information about this choice, including consent withdrawal, please see our [Privacy Policy](https://www.plos.org/privacy-policy).

Reviewer #1: No

Reviewer #2: No

[NOTE: If reviewer comments were submitted as an attachment file, they will be attached to this email and accessible via the submission site. Please log into your account, locate the manuscript record, and check for the action link "View Attachments". If this link does not appear, there are no attachment files.]

While revising your submission, please upload your figure files to the Preflight Analysis and Conversion Engine (PACE) digital diagnostic tool, <https://pacev2.apexcovantage.com/>. PACE helps ensure that figures meet PLOS requirements. To use PACE, you must first register as a user. Registration is free. Then, login and navigate to the UPLOAD tab, where you will find detailed instructions on how to use the tool. If you encounter any issues or have any questions when using PACE, please email PLOS at [figures@plos.org](mailto:figures@plos.org). Please note that Supporting Information files do not need this step.

*In compliance with data protection regulations, you may request that we remove your personal registration details at any time.* [*(Remove my information/details)*](https://www.editorialmanager.com/pone/login.asp?a=r)*. Please contact the publication office if you have any questions.*

**References:**

1. Eich, W. *et al.*, Z Rheumatol 2008;67:665-666, 668-672, 674-666.

2. Wolfe, F. *et al.*, Arthritis Care Res (Hoboken) 2010;62:600-610.

3. Wolfe, F. *et al.*, Arthritis Rheum 1990;33:160-172.

4. Radloff, L. S., Appl Psychol Meas 1977;3:385–401.

5. Zhang, Y. *et al.*, Ther Adv Neurol Disord 2021;14:17562864211007708.

6. Richter, D. *et al.*, Front Neurol 2018;9:645.
